# Interferon Gamma Activated Macrophages Kill Mycobacteria by Nitric Oxide Induced Apoptosis

**DOI:** 10.1371/journal.pone.0019105

**Published:** 2011-05-02

**Authors:** Susanne Herbst, Ulrich E. Schaible, Bianca E. Schneider

**Affiliations:** 1 Department of Immunology and Infection, Faculty of Infectious and Tropical Diseases, London School of Hygiene & Tropical Medicine, London, United Kingdom; 2 Department of Molecular Infection Biology, Research Center Borstel, Borstel, Germany; Institut Pasteur, France

## Abstract

*Mycobacterium tuberculosis* is an intracellular pathogen of macrophages and escapes the macrophages' bactericidal effectors by interfering with phagosome-lysosome fusion. IFN-γ activation renders the macrophages capable of killing intracellular mycobacteria by overcoming the phagosome maturation block, nutrient deprivation and exposure to microbicidal effectors including nitric oxide (NO). While the importance about NO for the control of mycobacterial infection in murine macrophages is well documented, the underlying mechanism has not been revealed yet. In this study we show that IFN-γ induced apoptosis in mycobacteria-infected macrophages, which was strictly dependent on NO. Subsequently, NO-mediated apoptosis resulted in the killing of intracellular mycobacteria independent of autophagy. In fact, killing of mycobacteria was susceptible to the autophagy inhibitor 3-methyladenine (3-MA). However, 3-MA also suppressed NO production, which is an important off-target effect to be considered in autophagy studies using 3-MA. Inhibition of caspase 3/7 activation, as well as NO production, abolished apoptosis and elimination of mycobacteria by IFN-γ activated macrophages. In line with the finding that drug-induced apoptosis kills intracellular mycobacteria in the absence of NO, we identified NO-mediated apoptosis as a new defense mechanism of activated macrophages against *M. tuberculosis*.

## Introduction

Macrophages are important effector cells in immunity to intracellular bacteria but at the same time are exploited as host cells by a number of microorganisms such as *Mycobacterium tuberculosis* (*M. tuberculosis*). Delivery of invading bacteria to phagolysosomes represents an important mechanism to eliminate intracellular pathogens by exposing them to cellular microbicides. In resting macrophages, pathogenic mycobacteria block phagosome maturation to assure intracellular survival and replication [1]–[3]. In contrast, IFN-γ activated macrophages are capable to overcome blockage of phagosome maturation, possibly through induction of and intersection with the autophagic pathway. Within the phagolysosome, mycobacteria are deprived of essential nutrients such as iron and exposed to microbicidal effectors generated by IFN-γ activated macrophages such as anti-microbial peptides (AMP) and reactive oxygen or nitrogen intermediates, the products of NADPH oxidase and nitric oxide synthase (NOS2), respectively [4]–[6].

Autophagy has been identified as effector mechanism associated with inflammatory T helper 1 (Th1) immune responses induced by the Th1 hallmark cytokine, IFN-γ. Killing of intracellular mycobacteria by IFN-γ activated macrophages has been linked to autophagy [5] whereas alternative activation by the Th2 cytokines IL-4 and IL-13 counter regulates autophagy and promotes mycobacterial growth probably by induction of arginase 1 and depletion of arginine, the substrate shared with NOS2 [7]–[9].

In the murine model of *M. tuberculosis* infection, the importance of nitric oxide (NO) for the control of mycobacterial growth is well established [10]. NOS2-deficient mice are more susceptible to *M. tuberculosis* infection than wild type ones [11]. In human macrophages however, the role of NO in the control of mycobacterial infections is less clear. It should be noted, that apoptosis has been associated with reduced mycobacterial viability in human macrophages [12]. Apoptosis is a tightly regulated mechanism leading to programmed cell death and is essential during development and tissue homeostasis [13], [14]. Although different apoptotic pathways have been described, major players in induction and execution are caspases. Apoptosis can be triggered either extrinsically by ligation and dimerization of death domain containing receptors such as the tumor necrosis factor receptor resulting in the activation of caspase 8/10, or intrinsically by cellular stress such as DNA damage, growth factor withdrawal or exposure to free radicals resulting in mitochondrial outer membrane permeability, cytochrome c release and activation of caspase 9. These inducer capases subsequently initiate a cascade by cleaving the so-called effector caspases, i.e. caspase-3, -6 and -7. This causes disruption of intracellular transport and signal transduction, finally resulting in the disintegration of the cell into apoptotic bodies [15]. NO has been described as an inducer of apoptosis in macrophages via a caspase/cytochrome c dependent mechanism [16]–[19].

Direct susceptibility of mycobacteria to reactive nitrogen intermediates *in vitro* is strain-, dose- and time-dependent [20]–[22]. However, the mechanism by which NO mediates killing of mycobacteria by activated macrophages, be it by direct toxicity, indirect interference with mycobacterial virulence [23] or by acting as a second messenger [24], is still a matter of debate.

In the present study we demonstrate that IFN-γ induces apoptosis in mycobacteria-infected macrophages in an NO-dependent manner. Both, prevention of apoptosis by inhibiting NO production as well as blockade of the pro-apoptotic caspase cascade abolished subsequent killing of mycobacteria, while autophagy was not involved in this process. Together with the finding that drug-induced apoptosis kills intracellular mycobacteria in the absence of NO, our results demonstrate that in INF-γ activated macrophages, NO facilitates its antimicobacterial action by the induction of host cell apoptosis.

## Materials and Methods

### Ethics statement

Mice bred under specific pathogen free conditions at the Biological Service Facility of the London School of Hygiene and Tropical Medicine (London, UK), and bone marrow cells were isolated after mice were put to death. The procedures used were according to the UK Home Office regulations in accordance with the Animals (Scientific Procedures) Act 1986, approved by the LSHTM Ethics Review Committee and performed under the Home Office Animal Project Licence No. 70/6934.

### Bacterial strains and culture

*M. tuberculosis* H37Rv (*M. tuberculosis*) and *M. bovis* BCG (BCG) Pasteur were grown in Middlebrook 7H9 broth (BD Biosciences, UK) supplemented with 0.5% glycerol and Tween 80, respectively, and OADC enrichment medium (BD). Bacterial cultures were harvested and aliquots were stored at −80°C until later use. Viable cell counts in thawed aliquots were determined by plating serial dilutions of cultures onto Middlebrook 7H11 agar plates followed by incubation at 37°C.

### Cell culture and *in vitro* infection

Bone marrow cells from C57BL/6 or TLR2/4/9 KO mice were harvested and differentiated to macrophages in DMEM containing 20% L-cell supernatant, 10% heat-inactivated FCS, 5% heat-inactivated HS, and 2 mM glutamine for 6 days. The murine macrophage-like cell line RAW 264.7 was used for transfection experiments. Raw cells were cultured in DMEM containing 10% heat-inactivated FCS and 2 mM glutamine at 37°C, 5% humified CO_2_. Cells were passaged every 4 days 1∶8 or 1∶3 one day before transfection. Cells were cultured in antibiotic-free medium at all times.

To analyze the anti-bacterial activity of bone marrow-derived macrophages (BMMΦ) they were left unstimulated or stimulated with recombinant IFN-γ (2000 U/ml, R&D Systems) over night (O/N), and infected with *M. tuberculosis* or BCG at a multiplicity of infection of 1∶1 (*M. tuberculosis*) or 1∶10 (BCG) for 1 h. Cells were washed to remove extracellular bacteria and either lysed with 0.5% v/v Triton X-100 in H_2_O or further incubated in fresh medium containing IFN-γ in combination with 3-methyladenine (3-MA, 10 mM, Sigma-Aldrich), N^G^-methyl-l-arginine (NMLA, 1 mM, Calbiochem), wortmannin (WM, 100 nM, Sigma-Aldrich), IL-4 (50 U/ml, R&D Systems), pan caspase inhibitor Z-VAD-FMK (20 µM, Promega), caspase 3/7 inhibitor Ac-DEVD-CHO (100 µM, Promega), or S-nitroso-N-acetylpenicillamine (SNAP, 300 µM, Calbiochem). Staurosporine (1 µM, Sigma-Aldrich) was used for induction of apoptosis. Hanks balanced salt solution (HBSS, Gibco) or rapamycin (50 µg/ml, Sigma-Aldrich) were used to induce autophagy. BMMΦ were lysed with 0.5% v/v Triton X-100 in H_2_O at the indicated times and serial dilutions were plated onto Middlebrook 7H11 agar plates followed by incubation at 37°C.

### Transfection and fluorescence microscopy

RAW264.7 cells were transfected with an LC3-eGFP encoding plasmid (kindly provided by T. Yoshimori [25]) using the transfection reagent Turbofect™ (Fermentas) according to the manufacturer's protocol. Briefly, 2×10^5^ cells/well were seeded in a 24 well plate and plasmid DNA (2 µg DNA; GenElute endotoxin-free plasmid midiprep kit, PLED-35, Sigma) together with 2 µl Turbofect™ per well was added. The next day, the medium was changed and cells were infected as described below.

For microscopy, LC3-eGFP expressing RAW264.7 cells were stimulated with recombinant IFN-γ (2000 U/ml) for 6 h and infected with BCG at a MOI 10 for 1 h. Cells were washed to remove extracellular bacteria and further incubated in fresh medium with or without IFN-γ (2000 U/ml). Cells were fixed in 4% paraformaldehyde for 30 min at RT and mounted in Vectashield ® hard mounting medium containing DAPI (Vector Laboratories) for analysis by confocal microscopy (Zeiss LSM 510).

### Nitric oxide assay

Nitric oxide was determined in supernatants of BMMΦ cultures as NO_2_^−^ following reduction of NO_3_^−^, using the Griess-reaction described previously [26].

### Assessment of apoptosis

The induction of apoptosis was determined by measuring caspase 3/7 activity which was assessed with the Apo-ONE® Homogeneous Caspase-3/7 Assay (Promega) according to the manufacturer's protocol. In brief, cells were cultured in a 96 well plate in a volume of 100 µl, infected and treated as described before. To quantify caspase activity, 100 µl assay reagent were added per well and after 5 h, fluorescence was measured at 520 nm. To enumerate apoptotic cells, binding of annexin V-PE to plasma membranes of apoptotic BMMΦ was analysed by FACS.

### Statistical analysis

Statistical analysis was performed by students *t*-test or by ANOVA followed by the Tukey's multiple comparison test defining different error probabilities as significant (* *p*≤0.05), (** *p*≤0.01), (*** *p*≤0.001).

## Results

### IFN-γ mediated killing of intracellular mycobacteria is inhibited by 3-MA and NMLA

**Figure 1:**
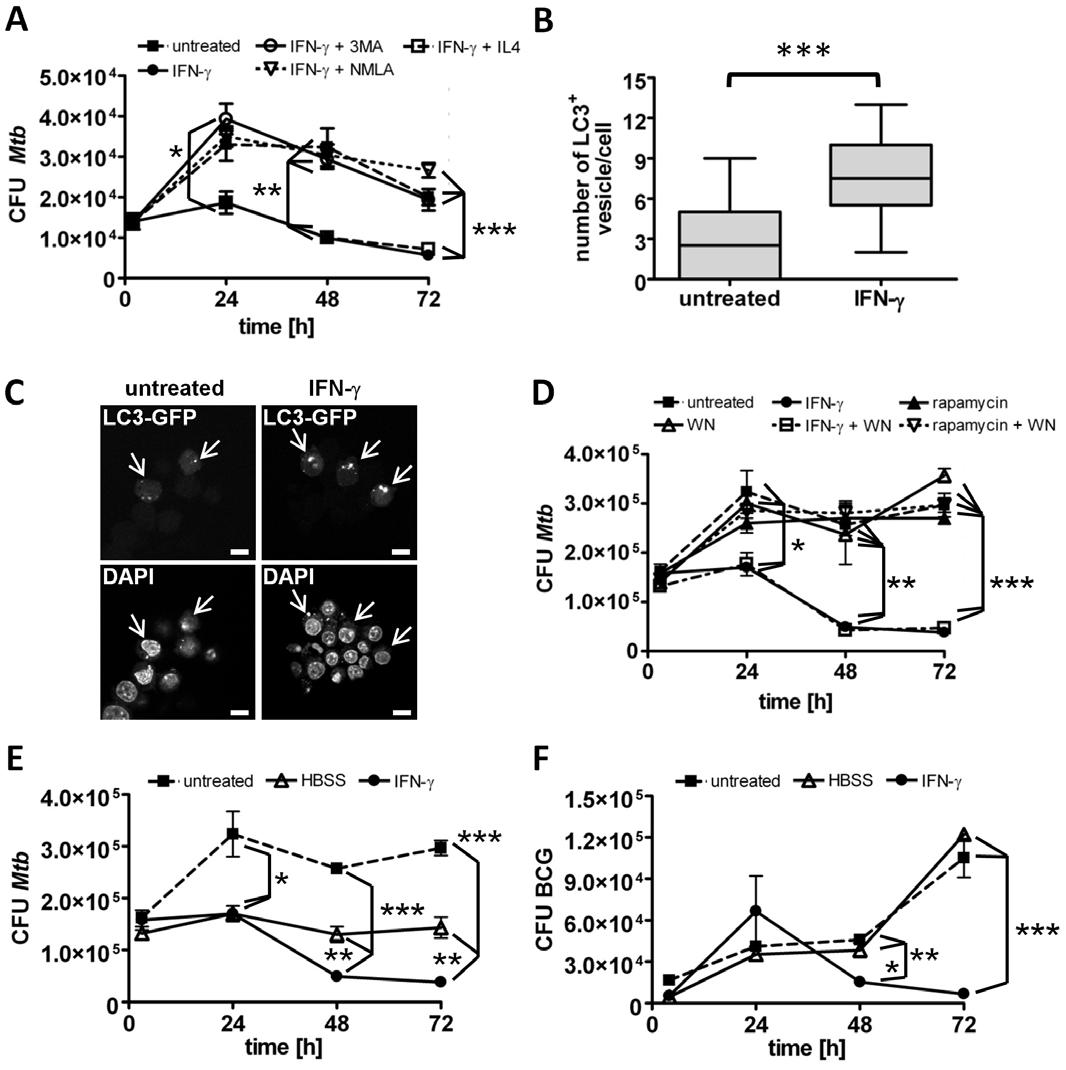
Autophagy does not affect mycobacterial survival in BMMΦ. Resting or IFN-γ activated (2000 U/ml O/N) BMMΦ were infected with *M. tuberculosis* (MOI 1) or BCG (MOI 10) for 1 h, and subsequently treated with 3-MA (10 mM), NMLA (1 mM), IL-4 (50 U/ml), rapamycin (50 µg/ml), wortmannin (WM, 100 nM) or hanks balanced salt solution (HBSS). To determine mycobacterial survival, macrophages were lysed every 24 h and serial dilutions were plated onto agar plates for CFU determination (A, D–F). Data represent means ± SD of triplicate cultures from one representative experiment out of three. Statistical analysis was performed by ANOVA (* *p*<0.05; ** *p*<0.01; *** *p*<0.001). B and C) *Untreated or* IFN-γ *activated* (2000 U/ml for 6 h) RAW 264.7 cells expressing eGFP-LC3 were analyzed by confocal microscopy for the number of LC3-positive vesicles 4 h post infection with BCG (MOI 10). Corresponding cells are labeled with arrows. B) Data from one out of two independent experiments are shown as means ± SD (n = 30 cells). Statistical analysis was performed by students *t*-test (*** *p*<0.001). Representative confocal images are shown in C. Scale bar 10 µm.

Both, NO production and autophagy were proposed as key functions in IFN-γ mediated killing of intracellular mycobacteria by murine macrophages. In order to analyse their role in growth restriction of intracellular mycobacteria over a time course of several days, we infected resting or IFN-γ activated (2000 U/ml O/N) bone marrow derived macrophages (BMMΦ) with *M. tuberculosis* or BCG for 1 h. Subsequently, inhibitors of autophagy or NOS2, 3-MA (10 mM) and NMLA (1 mM), respectively, or the Th2 cytokine IL-4 (50 U/ml) were added. After the time points indicated, BMMΦ were lysed and plated in serial dilutions onto 7H11 agar plates for CFU determination. Both 3-MA and NMLA but not IL-4 inhibited killing of intracellular *M. tuberculosis* (Fig. 1 A) and BCG (data not shown), respectively, indicating that both, autophagy and NO, contribute to the mycobactericidal activity of IFN-γ activated macrophages. The inhibitors were tested for cytotoxicity using the WST-1 cell proliferation reagent. NMLA and 3-MA did not affect cell viability over the time course of the experiment (Fig. S1 A and B).

### Autophagy is not sufficient to restrict growth of intracellular mycobacteria

In order to confirm autophagy induction by IFN-γ we counted newly formed LC3-positive autophagosomes in IFN-γ activated BCG infected LC3-eGFP expressing Raw macrophages. LC-3 specifically translocates from the cytosol to forming autophagosomal membranes. As shown before [5], IFN-γ treatment resulted in a significant increase in LC3 positive vesicles per cell (Fig. 1 B and C). To reveal whether autophagy does influence mycobacterial survival inside macrophages over a time course of several days, we induced autophagy by rapamycin (50 µg/ml), and used wortmannin (WM, 100 nM) as autophagy inhibitor on both IFN-γ and rapamycin treated macrophages. Notably, neither did rapamycin restrict replication of *M. tuberculosis* or BCG nor did WM abrogate IFN-γ induced killing (Fig. 1 D and data not shown). In line with this, autophagy induction by starvation did not kill *M. tuberculosis* although it impaired *M. tuberculosis* growth whereas BCG replication was not affected at all in starved BMMΦ (Fig. 1 E and F). Growth restriction of *M. tuberculosis* by starvation was most likely a result of nutrient deprivation rather than of autophagy as indicated by the failure of rapamycin to induce, and of WM to abrogate killing of *M. tuberculosis* (Fig. 1 D). Together, these results suggest that autophagy, though induced by IFN-γ activation does not target intracellular mycobacteria in these long-term assays and is most probably not the underlying mechanism of killing *M. tuberculosis* or BCG by IFN-γ activated macrophages.

### 3-MA inhibits NO-production

**Figure 2:**
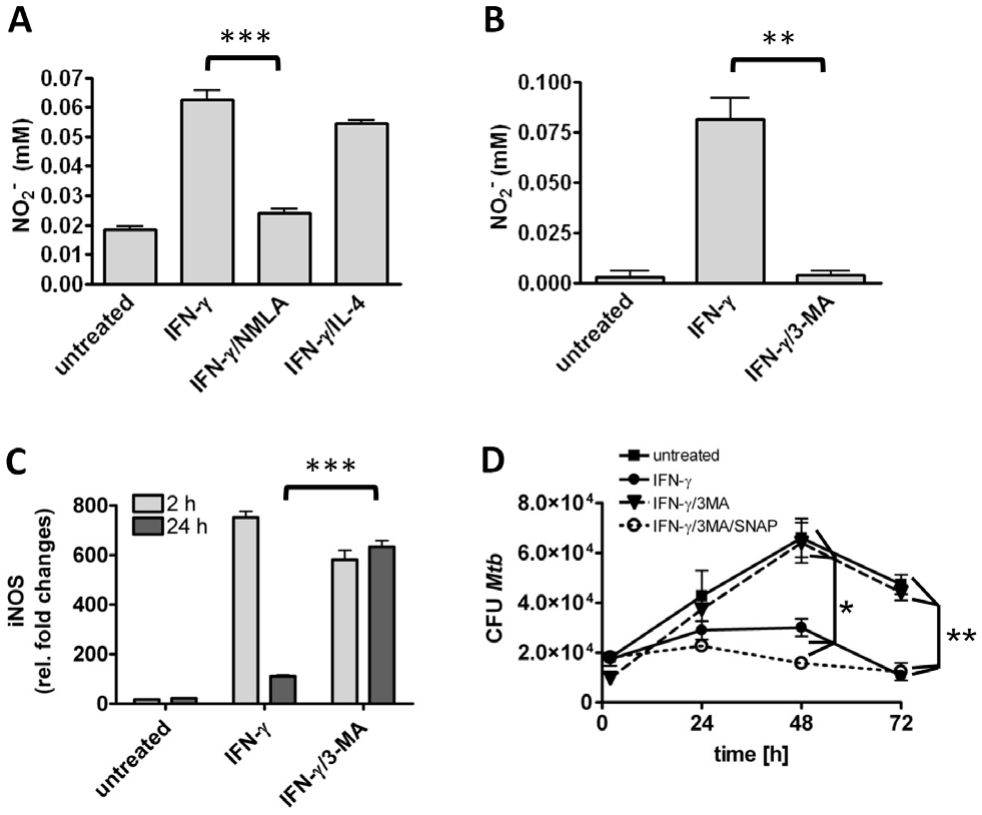
3-MA inhibits NO-production in IFN-γ activated BMMΦ. A and B) Resting or IFN-γ activated (2000 U/ml O/N) BMMΦ were infected with *M. tuberculosis* (MOI 1) for 1 h, and subsequently treated with NMLA (1 mM), IL-4 (50 U/ml), or 3-MA (10 mM). Supernatants were analyzed for the production of NO using the Griess assay. Note, that 3-MA treatment abrogated NO production (B). C) NOS-2 mRNA levels were analyzed by quantitative RT-PCR 2 h and 24 h post infection with BCG (MOI 10). D) Resting or IFN-γ activated (2000 U/ml O/N) BMMΦ were infected with *M. tuberculosis* (MOI 1) for 1 h and subsequently treated with 3-MA or 3-MA plus SNAP (300 µM). To determine mycobacterial survival, macrophages were lysed every 24 h and serial dilutions were plated onto agar plates for CFU determination. Data represent means ± SD of triplicate cultures from one representative experiment out of 2 (C) or three (A, B and D), respectively. Statistical analysis was performed by ANOVA (* *p*<0.05; ** *p*<0.01; *** *p*<0.001).

3-MA is described as specific inhibitor of autophagy and abrogated the anti-mycobactericidal activity of IFN-γ activated BMMΦ. However, our data shown above (Fig. 1 B–D) do indicate that the inhibitory activity of 3-MA was independent of autophagy. In good agreement, but in contrast to what has been published before [7] IL-4 was also not able to counteract the IFN-γ mediated killing of intracellular mycobacteria (Fig. 1 A) suggesting that autophagy does not contribute to the anti-mycobacterial activity of IFN-γ activated BMMΦ in long-term culture systems analysed herein. IFN-γ rendered macrophages able to kill mycobacteria in a NO-dependent manner over a concentration range between 20 and 2000 U/ml. Even at low IFN-γ concentrations of 20 U/ml, IL-4 failed to prevent IFN-γ mediated killing of *M. tuberculosis* (Figure S2). Assessment of NO production in supernatants from *M. tuberculosis* or BCG infected BMMΦ revealed that IL-4 was not able to limit NO production (Fig. 2 A and data not shown). However and to our great surprise, 3-MA almost completely abrogated NO production by infected IFN-γ activated BMMΦ (Fig. 2 B and data not shown) indicating that inhibition of IFN-γ mediated killing of mycobacteria by 3-MA is a result of blocking NO production. Of note, 3-MA did not inhibit expression of NOS2 but even resulted in prolonged expression compared to macrophages treated with IFN-γ alone (Fig. 2 C). To reveal whether 3-MA inhibits IFN-γ induced killing of mycobacteria by directly preventing NO production, the NO donor SNAP (S-nitroso-N-acetylpenicillamine) was added simultaneously with 3-MA, and *M. tuberculosis* or BCG growth was monitored over time. Addition of SNAP restored the macrophage ability to kill ingested mycobacteria in the presence of 3-MA (Fig. 2 D and data not shown). These data strongly indicate that the inhibitory effect of 3-MA was due to blocking NO production rather than by inhibiting autophagy.

### NO induced apoptosis is responsible for the killing of intracellular mycobacteria in IFN-γ activated macrophages

**Figure 3:**
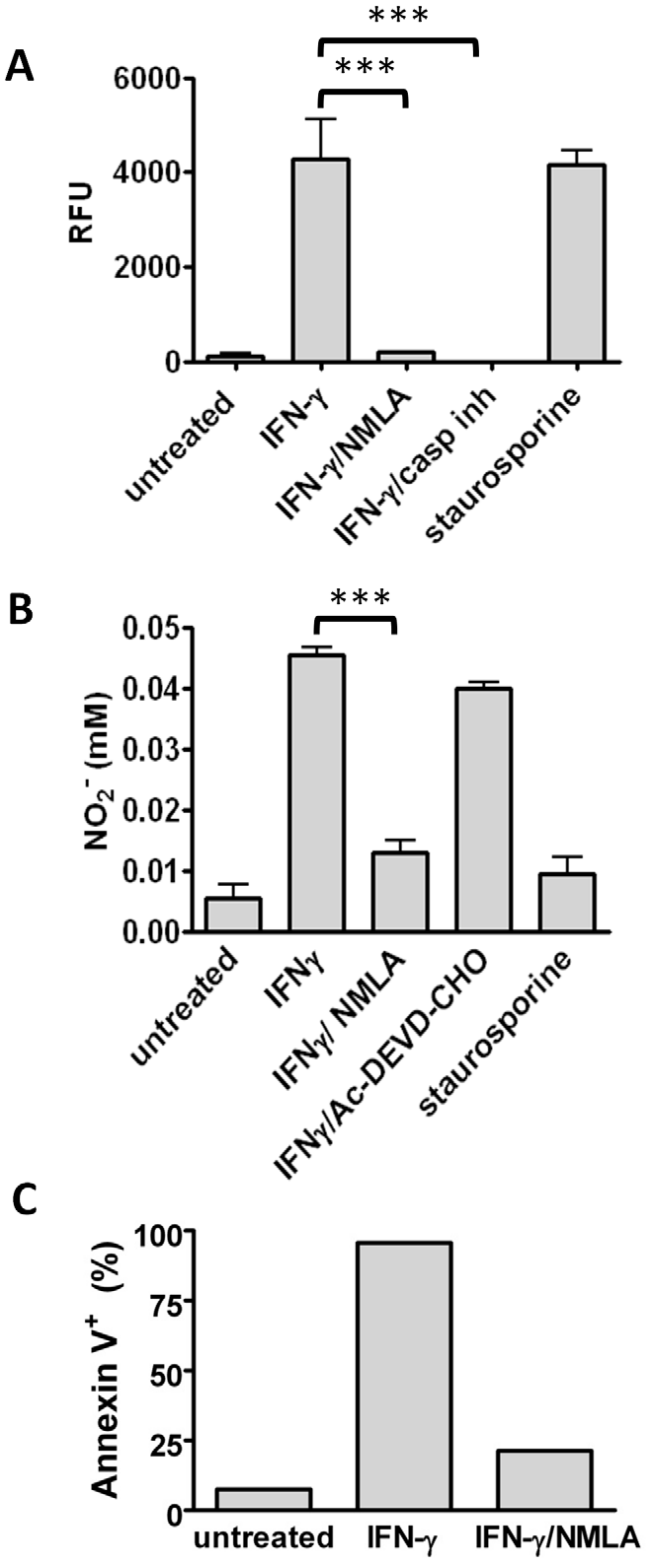
IFN-*γ* induces apoptosis in BMMΦ in an NO-dependent manner. Resting or IFN-γ activated (2000 U/ml O/N) BMMΦ were infected with BCG (MOI 10, A and B) or *M. tuberculosis* (MOI 1, C) for 1 h, and subsequently treated with NMLA (1 mM), caspase 3/7 inhibitor (Ac-DEVD-CHO, 100 µM; Z-VAD, 20 µM), or staurosporine (1 µM). A) Caspase 3/7 activity 48 h p.i. with BCG is shown as relative fluorescence units (RFU) of the non-fluorescent substrate Z-DEVD-R110 which becomes fluorescent upon cleavage. Data from one representative experiment out of three are shown as means ± SD from triplicate cultures. Statistical analysis was performed by ANOVA (*** *p*<0,001). B) NO was measured in cell culture supernatants 48 h p.i. Data from one representative experiment out of three are shown as means ± SD from triplicate cultures. Statistical analysis was performed by ANOVA (*** *p*<0,001). C) Annexin V-PE binding to BMMΦ 48 h post *M. tuberculosis* infection was determined by FACS. Data are shown as mean % of annexin V-positive cells from duplicate cultures.

It has been shown that NO is able to induce caspase-dependent apoptosis [19], [27]–[29] in macrophages. Host cell apoptosis however, has also been described to cause death of intracellular mycobacteria [12], [30]. In order to analyse whether NO induces apoptosis in IFN-γ activated and mycobacteria infected BMMΦ, we determined NO production and concomitant induction of apoptosis in infected BMMΦ. To do so, resting or IFN-γ activated BMMΦ were infected with BCG for 1 h, and subsequently treated with NMLA (1 mM) or Z-VAD (20 µM). Staurosporine (1 µM) was used as positive control to induce caspase-mediated apoptosis. Apoptosis was determined by measuring caspase 3/7 activity using the substrate Z-DEVD-R110. 48 h post BCG infection, IFN-γ treated BMMΦ showed increased caspase-3 activity (Fig. 3 A) in a comparable amount as measured in staurosporine-treated BMMΦ. In contrast, the NOS2 inhibitor NMLA blocked IFN-γ induced caspase activation nearly as efficient as the caspase inhibitor, demonstrating that NO is responsible for IFN-γ induced apoptosis (Fig. 3 A). To determine the number of apoptotic BMMΦ upon IFN-γ activation and *M. tuberculosis* infection, binding of annexin V-PE to phosphatidylserine was determined by FACS analysis. Around 90% of IFN-γ activated BMMΦ stained positive for annexin V 48 h post infection, whereas only 25% of the cells became annexin V positive when NO production was blocked (Fig. 3 C). Uninfected BMMΦ did not bind annexin V-PE upon IFN-γ treatment (data not shown). Without infection, IFN-γ activated BMMΦ produce very little amounts of NO (<0.01 mM). These data clearly show that induction of apoptosis in activated mycobacteria infected macrophages depends on NO production.

**Figure 4:**
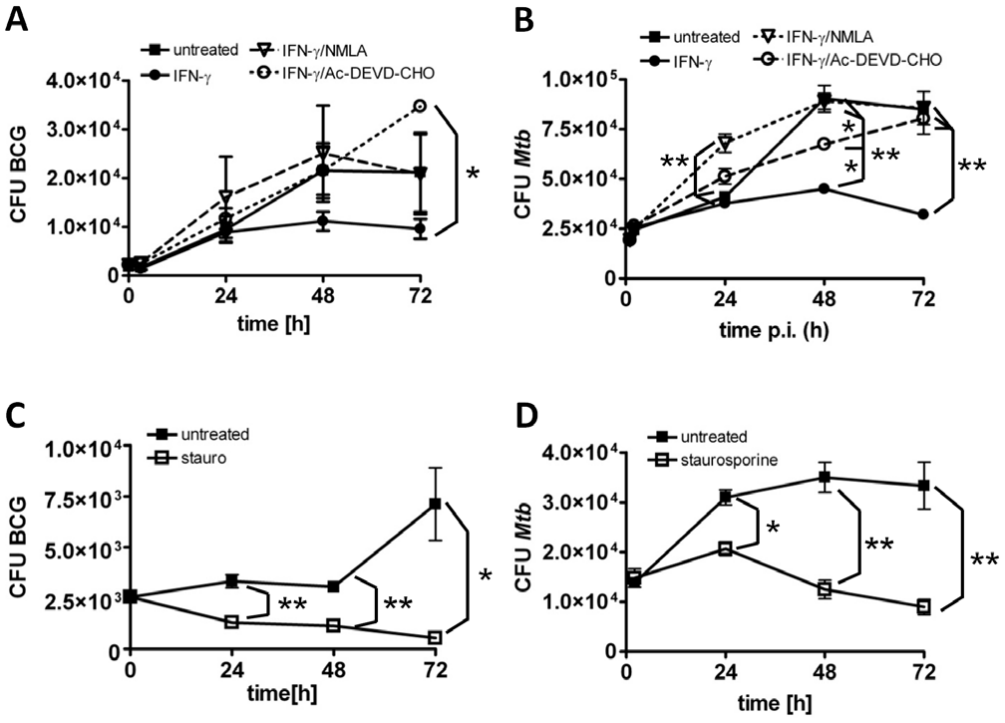
NO induced apoptosis is responsible for the growth restriction of intracellular mycobacteria in IFN-γ activated macrophages. Resting or IFN-γ activated (2000 U/ml O/N) B6 (A, C and D) or TLR2/4/9 KO (B) BMMΦ were infected with BCG (MOI 10; A, C) or *M. tuberculosis* (MOI 1; B, D) for 1 h, and subsequently treated with NMLA (1 mM) or caspase 3/7 inhibitor (Ac-DEVD-CHO, 100 µM). Staurosporine (1 µM) was used to induce apoptosis. To determine mycobacterial survival, macrophages were lysed every 24 h and serial dilutions were plated onto agar plates for CFU determination. Data from one representative experiment out of two, respectively, are shown as means ± SD of triplicate cultures. Statistical analysis was performed by ANOVA (* *p*<0.05; ** *p*<0.01).

In order to reveal whether apoptosis is responsible for IFN-γ mediated killing of intracellular mycobacteria, we treated infected IFN-γ activated BMMΦ with caspase inhibitors. As mentioned before, inhibition of NO production permitted intracellular replication of BCG (Fig. 4 A). More importantly, inhibition of apoptosis by a caspase 3/7 inhibitor also prevented IFN-γ mediated growth restriction and resulted in intracellular replication of BCG (Fig. 4 A). Of note, NO production was not affected by caspase-inhibitor treatment (Fig. 3 B), indicating that NO-induced apoptosis rather than NO itself restricted growth of intracellular mycobacteria. This result was corroborated by the observation that staurosporine treatment, which by itself did not trigger production of NO (Fig. 3 C), resulted in efficient killing of both, BCG and *M. tuberculosis* (Fig. 4 C and D).

While treatment of IFN-γ activated BMMΦ with a caspase-inhibitor permitted intracellular growth of BCG, the same inhibitor was cytotoxic to *M. tuberculosis* infected macrophages (not shown). Interestingly, when infected and IFN-γ activated TLR2/4/9 KO BMMΦ, which were also able to restrict mycobacterial propagation [31], were treated with a caspase 3/7 inhibitor, intracellular growth of *M. tuberculosis* was rescued (Fig. 4 B) without BMMΦ succumbing to death by inhibitor treatment. Taken together, our data demonstrate that killing of mycobacteria by IFN-γ activated macrophages is due to NO- mediated induction of apoptosis.

## Discussion

One strategy for defense against intracellular pathogens is to activate the programmed death of the host cell. Thereby, the pathogen is deprived of its protective niche for survival and replication. It has been shown that avirulent mycobacteria induce apoptosis in resting murine and human macrophages while virulent strains do not or even inhibit programmed cell death indicating maintenance of host cell integrity as measure to secure the intracellular niche and to avoid immune recognition [32]–[39]. The role of apoptosis as an innate defense mechanism against mycobacterial infection has only been studied in resting macrophages. Since NO is known to induce apoptosis in macrophages [18], [19], [28], [40] and IFN-γ is a potent inducer of NOS2 expression in murine macrophages, we hypothesized that apoptosis is an IFN-γ mediated effector mechanism against intracellular mycobacteria. It is well established that NO is, at least in mice one of the most important IFN-γ induced anti-mycobacterial effectors of macrophages, but the killing mechanism has not been revealed. NO could either affect bacterial viability directly by affecting multiple targets such as mycobacterial DNA and copper metabolism [41]–[43], target mycobacterial virulence factors [23] or act as second messenger [24].

In the present study we could show that IFN-γ activated BMMΦ undergo apoptosis after BCG or *M. tuberculosis* infection. Apoptosis was strictly dependent on NO production since inhibition of NOS2 activity by NMLA blocked apoptosis. More importantly, we could show that apoptosis was responsible for the IFN-γ mediated killing of intracellular mycobacteria since inhibition of the executor caspases 3 and 7 permitted survival and replication of BCG in IFN-γ activated BMMΦ despite concomitant production of NO. It has previously been shown that IFN-γ and LPS treated murine macrophages undergo apoptosis in an NO dependent manner [40], [44]. However, this has never been described as a defense mechanism against intracellular bacteria. Perfetto et al. [45] showed increased induction of NO-mediated apoptosis in *H. pylori* infected gastric adenocarcinoma cells upon IFN-γ activation. In this case however, apoptosis promoted survival of the pathogen. In contrast, macrophage apoptosis has been described to mediate killing of obligate intracellular *Coxiella burnetti* in IFN-γ activated THP-1 cells [46]. However, in contrast to our study, apoptosis induction was independent of NO but mediated by TNF-α. The role of TNF-α has not been addressed in our study since TNF-α production by infected macrophages was similar between activated and non-activated cells (data not shown), and both, apoptosis and killing of mycobacteria was entirely abolished by NOS2 inhibition.

Treatment of IFN-γ activated *M. tuberculosis* but not BCG infected wild type BMMΦ with either the pan caspase inhibitor VAD or Ac-DEVD-CHO inhibiting caspases 3 and 7 resulted in host cell death. Induction of macrophage apoptosis by caspase inhibitors in the presence of TLR signals has been described before [47]. This report suggests that *Mtb* and BCG differ with respect to abundance of TLR ligands, which can cause distinct sensitivities to caspase inhibitor treatment. In line with this, BCG infection of IFN-γ and DEVD-treated BMMΦ in the presence of LPS caused death of BMMΦ (data not shown). Therefore, we used TLR2/4/9 deficient BMMΦ for *M. tuberculosis* infection. While IFN-γ activated TLR2/4/9 deficient BMMΦ were able to kill *M. tuberculosis*, caspase 3/7 inhibition with Ac-DEVD-CHO rescued replication of *M. tuberculosis*. These data clearly demonstrate that NO-induced apoptosis rather than a direct bactericidal effect of NO is responsible for the killing of mycobacteria in IFN-γ activated macrophages. Treatment of macrophages with staurosporine, which directly induces caspase 3/7 dependent apoptosis without triggering NO production, killed mycobacteria effectively and with a similar kinetic than did IFN-γ treatment, thereby further establishing apoptosis as an IFN-γ mediated defense mechanism.

Virulent mycobacteria have evolved mechanisms to actively evade host cell apoptosis, which points out the relevance of apoptosis in anti-mycobacterial defense. These evasion mechanisms include inhibition of plasma membrane repair, which is necessary for apoptosis to prevent secondary necrosis [32], induction of anti-apoptotic proteins [48] and secretion of soluble TNF-α receptor to neutralize TNF-α [35]. While apoptosis is regarded as a defense mechanism against intracellular mycobacteria, necrosis seems rather beneficial supporting survival and intracellular growth of mycobacteria while concomitantly causing inflammation [30], [49]. Due to the apoptosis-associated anti-inflammatory activity and cross priming of T cells, killing of mycobacteria by NO mediated host cell apoptosis represents a strategy to limit inflammation while promoting protective immunity [38], [39], [50]. While we could confirm the mycobactericidal effect of host cell apoptosis, the underlying mechanism by which apoptosis is linked to the killing of intracellular mycobacteria remains to be elucidated. It has been suggested that apoptosis is associated with alterations in the architecture of the vesicular system of the host cell, promoting phago-lysosomal fusion by overcoming mycobacterial blockade of phagosome maturation [30]. Anti-bacterial activities of lysosomal cysteine proteases cathepsin B and G either directly or through generating AMPs from ubiquitin or other protein substrates released during autophagy [51] do not seem to play a role in killing mycobacteria since inhibition of cathepsin activity did not abrogate IFN-γ induced killing of BCG nor *M. tuberculosis* (data not shown).

Chemical inhibitors, though essential tools to study cellular signalling events and autophagy, often have limited specificity [52]. Therefore, the failure of rapamycin to restrict the growth of intracellular mycobacteria does not totally rule out a role for autophagy in IFN-γ mediated killing of mycobacteria. However, the fact that wortmannin failed to block IFN-γ induced killing whereas inhibition of both, NO-production and apoptosis promoted mycobacterial growth suggests only a minor role of autophagy in anti-mycobacterial activities of IFN-γ activated macrophages. Although 3-MA was able to inhibit IFN-γ activated killing of mycobacteria, this process was independent of autophagy. More importantly, we established that 3-MA is a strong inhibitor of NO production. Inhibition of NO-production is a not yet recognized effect of 3-MA and needs to be considered when using this drug in autophagy studies. 3-MA is an inhibitor for PI3 kinases, which are critically involved in the regulation of NO production by activated macrophages [53]. NO production was significantly impaired in PI3 kinase-deficient macrophages upon stimulation with LPS and IFN-γ despite normal expression of NOS-2. This was caused by reduced amounts of the cofactor tetra-hydrobiopterin, a stabilizer of NOS-2 dimer formation required for NOS-2 activity. We also found normal expression of both, NOS-2 mRNA and protein levels in 3-MA treated macrophages. Hence, 3-MA may have impaired NOS-2 protein dimerization thereby affecting NO production in IFN-γ activated macrophages. The exact mechanism of 3-MA on NOS-2 needs to be addressed in future experiments. In conclusion, our data establish apoptosis as a not yet described IFN-γ mediated defense mechanism against intracellular mycobacteria associated with protective Th1 immunity. The capability of virulent *M. tuberculosis* to interfere with programmed cell death is reversed by IFN-γ triggering NO-dependent host cell apoptosis. Thereby, macrophages are no longer safe havens for mycobacteria but instead doom the pathogen to follow their own path to death.

## Supporting Information

Figure S1 **Cell viability is not affected by NMLA or 3-MA.** Resting or IFN-γ activated (2000 U/ml O/N) BMMΦ were infected with *M. tuberculosis* (MOI 1) for 1 h, and subsequently treated with NMLA (1 mM; A) or 3-MA (10 mM; B). Cell viability at 48 h p.i. was determined with the WST-1 cell proliferation assay. Cell viability is determined by the amount of formazan dye produced by metabolically active cells by cleavage of the substrate WST and measured spectrophotometrically at 450 nm.
(TIF)

Figure S2 **IL-4 does not revert IFN-γ mediated killing of Mtb.** Resting or IFN-γ activated (20 U/ml O/N) BMMΦ were infected with *M. tuberculosis* (MOI 1) 1 h, and subsequently treated with IL-4 (50 U/ml). To determine mycobacterial survival, macrophages were lysed every 24 h and serial dilutions were plated onto agar plates for CFU determination. Data represent means ± SD of triplicate cultures from one representative experiment out of three. Statistical analysis was performed by ANOVA (** *p*<0.01; *** *p*<0.001).
(TIF)
