# Supplementary figures and images for: Interferon Gamma Activated Macrophages Kill Mycobacteria by Nitric Oxide Induced Apoptosis
Source: PLoS One. 2011 May 2;6(5):e19105. doi: 10.1371/journal.pone.0019105 (PMC3085516; doi:10.1371/journal.pone.0019105)

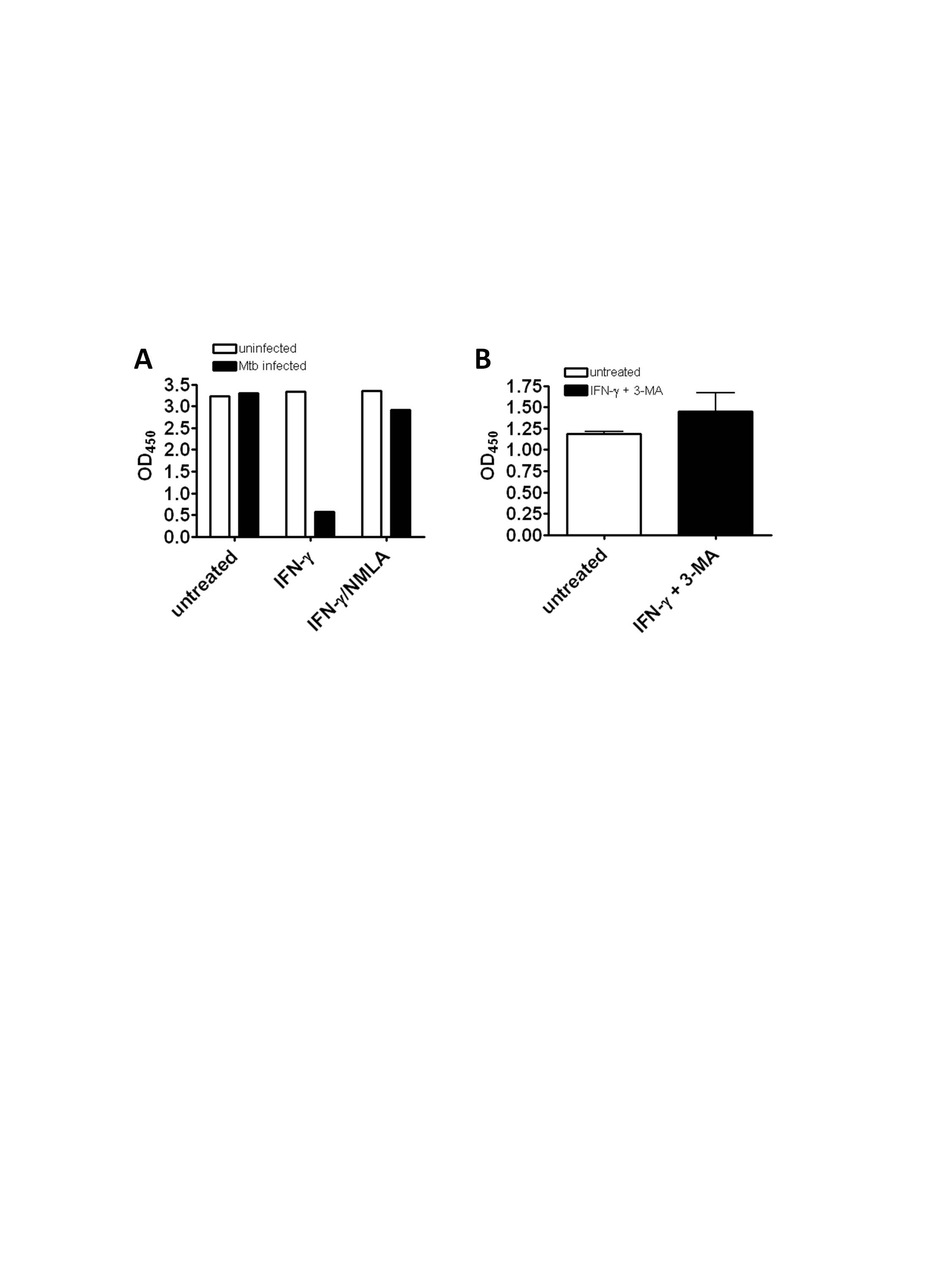

Supplement: Figure S1 — Cell viability is not affected by NMLA or 3-MA. Resting or IFN-γ activated (2000 U/ml O/N) BMMΦ were infected with M. tuberculosis (MOI 1) for 1 h, and subsequently treated with NMLA (1 mM; A) or 3-MA (10 mM; B). Cell viability at 48 h p.i. was determined with the WST-1 cell proliferation assay. Cell viability is determined by the amount of formazan dye produced by metabolically active cells by cleavage of the substrate WST and measured spectrophotometrically at 450 nm. (TIF) [file pone.0019105.s001.tif]

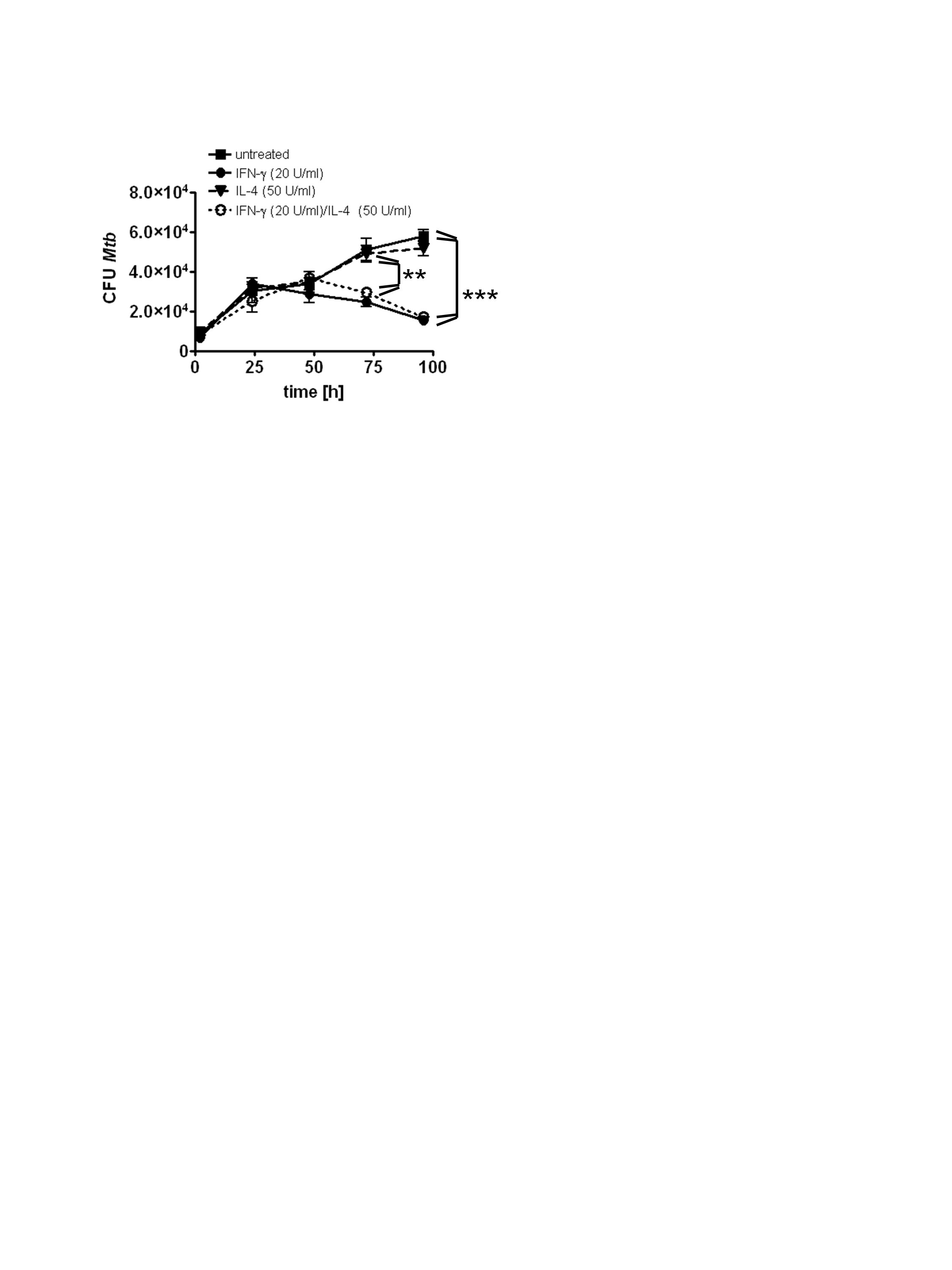

Supplement: Figure S2 — IL-4 does not revert IFN-γ mediated killing of Mtb. Resting or IFN-γ activated (20 U/ml O/N) BMMΦ were infected with M. tuberculosis (MOI 1) 1 h, and subsequently treated with IL-4 (50 U/ml). To determine mycobacterial survival, macrophages were lysed every 24 h and serial dilutions were plated onto agar plates for CFU determination. Data represent means ± SD of triplicate cultures from one representative experiment out of three. Statistical analysis was performed by ANOVA (** p<0.01; *** p<0.001). (TIF) [file pone.0019105.s002.tif]
